# Maguey, or Agave Americana; a Remedy for Scorbutus

**Published:** 1852-01

**Authors:** 


*Maguey, or Agave Americana; a Remedy for Scorbutus.*

Dr. Glover Perrin, U. S. A., has recorded in the September number of the New York Journal of Medicine, his observations on the use of this plant in scurvy. While stationed at Fort McIntosh, Texas, several soldiers of the regiment to which he was attached, were attacked with scurvy. Some were put as usual, upon the best of lemon juice, others upon citric acid, which treatment was continued for several days, with few, if any, signs of improvement. Being informed by the Curate of the town that he had once suffered from an attack in his own person, and had been cured by the use of domestic remedies, among which was the Maguey, Dr. Perrin determined to make trial of the expressed juice of this plant. A few days after its administration was begun, a decided amendment was observed, and all were soon relieved. The countenance, he said, so universally dejected and despairing in the patients affected with scurvy, is brightened up by contentment and hope in two days from the time of its introduction, and there was marked evidence of improvement at each successive visit. Dr. P. consequently places the Maguey far above lime-juice as a remedy in this disease. The plant grows indigenous in most parts of Texas, and, he was informed, of New Mexico and California. It grows in a sandy soil, and contains a large amount of vegetable and saccharine matter, and is of itself sufficiently nutritious to sustain a patient for days. The manner in which it is used, is as follows:—The leaves are cut off close to the root; they are placed in hot ashes until thoroughly cooked, when they are removed, and the juice expressed from them. The expressed juice is then strained, and may be used thus, or may be sweetened. It may be given in the dose of two to three ozs. three times daily.—*Ibid.*

